# Visualization of angiogenesis during cancer development in the polyoma middle T breast cancer model: molecular imaging with (*R*)-[^11^C]PAQ

**DOI:** 10.1186/2191-219X-4-17

**Published:** 2014-03-26

**Authors:** Erik Samén, Li Lu, Jan Mulder, Jan-Olov Thorell, Peter Damberg, Tetyana Tegnebratt, Lars Holmgren, Helene Rundqvist, Sharon Stone-Elander

**Affiliations:** 1 Department of Neuroradiology, Karolinska University Hospital, Solna, Stockholm 171 76, Sweden; 2 Department of Clinical Neuroscience, Karolinska Institutet, Solna, Sweden; 3 KERIC, Karolinska University Hospital, Solna, Stockholm 171 76, Sweden; 4 Science for Life Laboratory, Department of Neuroscience, Karolinska Institutet, Solna, Sweden; 5 Cancer Centrum Karolinska, Department of Oncology-Pathology, Karolinska Institutet, Solna, Sweden; 6 Department of Cell & Molecular Biology, Karolinska Institutet, Solna, Sweden

**Keywords:** PET, VEGFR2, Angiogenesis, [^11^C]PAQ, Cancer

## Abstract

**Background:**

Vascular endothelial growth factor receptor 2 (VEGFR2) is a crucial mediator of tumour angiogenesis. High expression levels of the receptor have been correlated to poor prognosis in cancer patients. Reliable imaging biomarkers for stratifying patients for anti-angiogenic therapy could therefore be valuable for increasing treatment success rates. The aim of this study was to investigate the pharmacokinetics and angiogenesis imaging abilities of the VEGFR2-targeting positron emission tomography (PET) tracer (*R*)-[^11^C]PAQ.

**Methods:**

(*R*)-[^11^C]PAQ was evaluated in the mouse mammary tumour virus-polyoma middle T (MMTV-PyMT) model of metastatic breast cancer. Mice at different stages of disease progression were imaged with (*R*)-[^11^C]PAQ PET, and results were compared to those obtained with [^18^ F]FDG PET and magnetic resonance imaging. (*R*)-[^11^C]PAQ uptake levels were also compared to *ex vivo* immunofluorescence analysis of tumour- and angiogenesis-specific biomarkers. Additional pharmacokinetic studies were performed in rat and mouse.

**Results:**

A heterogeneous uptake of (*R*)-[^11^C]PAQ was observed in the tumorous mammary glands. *Ex vivo* analysis confirmed the co-localization of areas with high radioactivity uptake and areas with elevated levels of VEGFR2. In some animals, a high focal uptake was observed in the lungs. The lung uptake correlated to metastatic and angiogenic activity, but not to uptake of [^18^ F]FDG PET. The pharmacokinetic studies revealed a limited metabolism and excretion during the 1-h scan and a distribution of radioactivity mainly to the liver, kidneys and lungs. In rat, a high uptake was additionally observed in adrenal and parathyroid glands.

**Conclusion:**

The results indicate that (*R*)-[^11^C]PAQ is a promising imaging biomarker for visualization of angiogenesis, based on VEGFR2 expression, in primary tumours and during metastasis development.

## Background

Vascular endothelial growth factor receptor 2 (VEGFR2) is a receptor tyrosine kinase that is a key mediator of vascular endothelial growth factor (VEGF)-stimulated proangiogenic signalling and is often overexpressed on activated endothelial cells in the tumours [1,2]. Several studies have implicated a close relationship between high VEGFR2 expression in tumours and invasive and metastatic behaviour, increased proliferation and poor patient prognosis [3-6]. For example, the tumour VEGFR2 expression in patients with invasive premenopausal breast cancer was found to be a prognostic marker for the response to oestrogen receptor-targeted therapy [7]. Anti-angiogenic therapy inhibits receptor signalling and has improved patient outcome in several types of cancer, e.g. glioblastoma and renal cell cancer. However, despite initially encouraging results, breast cancer studies have reported that anti-angiogenic treatment increases progression free survival but not overall survival compared to standard cytostatic treatment [8]. Finding reliable and accurate methods to individualize therapy is a high priority for cancer treatment of the future [9] and critical for increasing the overall benefit of anti-angiogenic therapy. Determining the extent to which these methods can identify and quantify molecular dysfunctions and therapeutic responses in preclinical models is a key step toward accelerating future translation to human studies.

We have previously reported promising positron emission tomography (PET) molecular imaging with the carbon-11-labelled VEGFR2-targeting tracer (*R*,*S*)-*N*-(4-bromo-2-fluorophenyl)-6-methoxy-7-((1-^11^C-methyl-3-piperidinyl)methoxy)-4-quinazolinamine ([^11^C]PAQ) [10]. Structurally, PAQ closely resembles the anti-angiogenic drug ZD6474 (Vandetanib) with exception for the chirality of the molecule and the reported 40 times stronger inhibitory properties (IC_50_ = 1 nM, *R*-isomer) for the VEGFR2, making it an attractive candidate as an imaging tracer [11]. The tracer demonstrated excellent *in vivo* stability and favourable pharmacokinetic behaviour. The uptake of radioactivity *in vivo* correlated well with *ex vivo* phosphor imaging and immunofluorescence (IF) performed postmortem, demonstrating high levels of radioactivity and VEGFR2, respectively, in the same tumour areas. The subcutaneous xenograft cancer provided important indications that [^11^C]PAQ could potentially be a valuable tracer with the ability to visualize regions with high VEGFR2 expression. Here, we have now developed a synthesis for the *R*-stereoisomer of the labelled tracer, based on the reported 10-fold lower IC_50_ value for the VEGFR2 and a 200-fold better selectivity versus the epidermal growth factor receptor for the *R-* compared to the *S*-isomer [11].

The ability of the carbon-11-labelled analogue of (*R*)-*N*-(4-bromo-2-fluorophenyl)-6-methoxy-7-((1-methyl-3-piperidinyl)methoxy)-4-quinazolinamine ((*R*)-[^11^C]PAQ) to detect levels of VEGFR2 *in vivo* is evaluated here in a transgenic mouse model of metastatic breast cancer, in which the expression of the polyoma middle T antigen (PyMT) oncoprotein is controlled by the mouse mammary tumour virus (MMTV). In the FVB genetic background, invasive mammary tumours and subsequent pulmonary metastases develop over 12 to 15 weeks. The model demonstrates gradual progression and significant stromal infiltration. It has been well characterized and shown to have a good translational potential [12]. Outgrowth of micrometastases in the MMTV-PyMT model has been shown to be dependent on endothelial progenitor cell infiltration followed by a distinct growth acceleration which correlated with an increased vascularization [13]. These features make this model attractive for evaluating novel PET radiotracers in a preclinical imaging setting. In this study, the MMTV-PyMT animals were injected with (*R*)-[^11^C]PAQ and scanned with PET at the age of 11 to 15 weeks when the presence of primary tumours had been confirmed by palpation and metastatic events in lung tissue could be expected.

Our goal was to evaluate the capability of (*R*)-[^11^C]PAQ to stratify levels of VEGFR2 during cancer development in the MMTV-PyMT model. The PET imaging results were compared to *ex vivo* phosphor imaging (PI) of malignant tissue combined with immunofluorescence (IF) analyses of several molecular components involved in angiogenesis and metastatic spread, including the VEGFR2. We also compare the uptake patterns of (*R*)-[^11^C]PAQ with 2-[^18^ F]-fluoro-2-deoxy-d-glucose ([^18^ F]FDG) PET in tumour and lung tissue. In addition to the tumour studies, the biodistribution and metabolism of (*R*)-[^11^C]PAQ were assessed.

## Methods

### Synthesis of radiotracers

**Figure 1:**
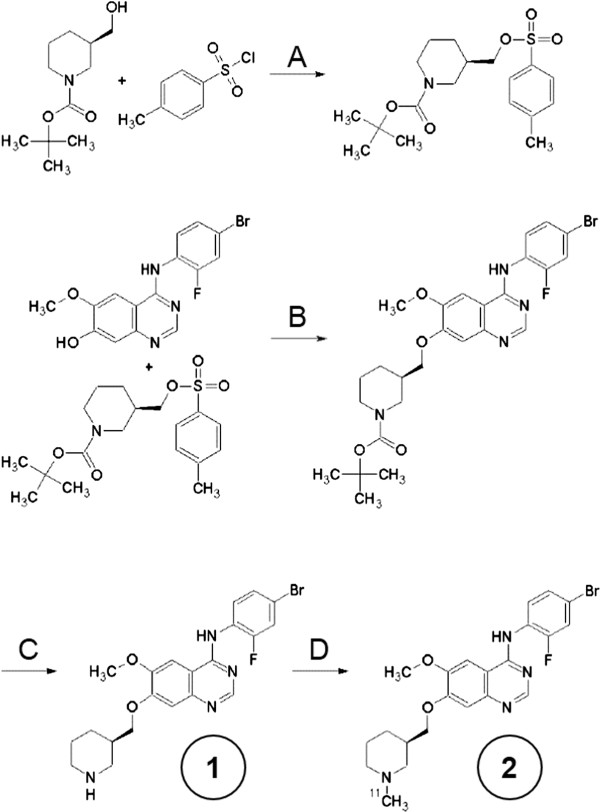
**Synthesis of precursor and radiolabeling. (A)** Triethylamine, dichloromethane, 23°C, 2 h. **(B)** K_2_CO_3_, dimethylformamide, 95°C, 2 h. **(C)** Trifluoroacetic acid, dichloromethane, 23°C, 3 h. **(D)** [^11^C]CH_3_I, K_2_CO_3,_ dimethylformamide, 95°C, 5 min.

A more detailed description of the synthesis of the precursor and radiolabelling can be found in Additional file 1. (*R*)-[^11^C]PAQ was produced according to Figure 1. The *N*-desmethyl (*R*)-precursor #1 was synthesized with an enantiomeric purity of >99%. Radiolabeling was accomplished using [^11^C]methyliodide prepared by methods first described in [14]. (*R*)-[^11^C]PAQ, #2, so prepared had a radiochemical purity of >98% and a specific activity typically of 1,000 to 2,000 GBq/μmol at injection. Decay-corrected radiochemical yield was approximately 10% at the end of synthesis. [2-^18^ F]-2-Fluoro-2-deoxy-d-glucose ([^18^ F]FDG), was obtained from batches made for clinical PET and had passed standard quality controls for Fludeoxyglucose (^18^ F) Karolinska.

### Animals and treatment

All experiments involving animals were conducted according to the research plan and regulations of the Karolinska Institutet and were approved by the local laboratory animal ethics committee (N325/09 with addendums N85/11 and N65/12). At the time of the study, the MMTV-PYMT mice (*n* = 11, 19 to 28 g, 11 to 15 weeks) had palpable tumours in at least one of the mammary glands. The female wild-type (WT) controls (*n* = 3, 18 to 23 g, 13 to 15 weeks) were healthy siblings to the MMTV-PyMT mice. The male Sprague-Dawley rats (Scanbur, Sollentuna, Sweden) (*n* = 3) used for metabolite assays and biodistribution experiments weighed 420 to 421 g and were 15 to 17 weeks old. Animals were housed under standard conditions according to local regulations with food and water *ad lib.*

### Metabolite assay

The rat was anaesthetized and placed on a heating pad (37°C) on the operating table. A PE-50 catheter was inserted into the femoral artery, and blood samples (300 μl) were collected via the catheter at 30 and 60 min after i.v. injection of 62.5 MBq of (*R*)-[^11^C]PAQ. The animal was sacrificed 60 min post-injection, and urine was collected from the bladder.

Blood and urine samples were added to plastic tubes containing heparin (50 μl, 5 IU/ml) and centrifuged (950 *g*, 2 min). The supernatant was transferred to a new tube and acetonitrile (200 μl) was added; the tube was then vortexed for a few seconds to mix and allow precipitation of proteins before centrifugation (950 *g*, 2 min). The resulting protein-free supernatant was injected on the HPLC system, and 20-s fractions were collected and measured in a gamma counter (1480 Wizard 3", PerkinElmer, Waltham, MA, USA).

The liquid chromatography (LC) system used for analysis of blood and urine samples consisted of a LC pump (Shimadzu LC-10 AD, Shimadzu, Kyoto, Japan), a μ-Bondapak C18 column (300 × 7.8 mm, 10 μm (Waters Corporation, Milford, MA, USA); mobile phase CH_3_CN:0.05 M NH_4_OAc 70:30 *v*/*v*; 7 ml/min) and a UV detector (Shimadzu) (*λ* = 254 nm) in series with a Beckman model 170 radiodetector (Beckman Coulter, Brea, CA, USA). Shimadzu Class VP software was used for the processing of the LC data.

### PET and MRI imaging

*In vivo* PET investigations were performed using a microPET Focus 120 scanner (CTI Concorde Microsystems, Knoxville, TN, USA). In the biodistribution assay, the rat was scanned using a continuous bed motion protocol to generate a whole-body image dataset. The MMTV-PyMT mice and controls were placed with the whole body in the field of view (7.68 cm). List mode data, collected continuously over 60 min (40 min for ^18^ F-FDG) starting at time of injection, were reconstructed by standard 2-D filtered back projection using a ramp filter. The matrix size of the reconstructed images was 256 × 256 × 95 with a spatial resolution of 1.3 mm. Data were normalized and corrected for randoms, dead time and radioactive decay. PET data were processed using MicroPET Manager and evaluated using the Inveon Research Workplace (IRW) software (Siemens Medical Systems, Malvern, PA, USA). (*R*)-[^11^C]PAQ and [^18^ F]FDG were administered via the tail vein (maximum 200 μl in mice and 1,000 μl in rat). Doses of (*R*)-[^11^C]PAQ injected ranged from 0.10 to 0.38 MBq/g in mice, and the rats used in the biodistribution assays received doses of 0.15 and 0.024 MBq/g of (*R*)-[^11^C]PAQ and (*R*,*S*)-[^11^C]PAQ, respectively.

**Figure 2:**
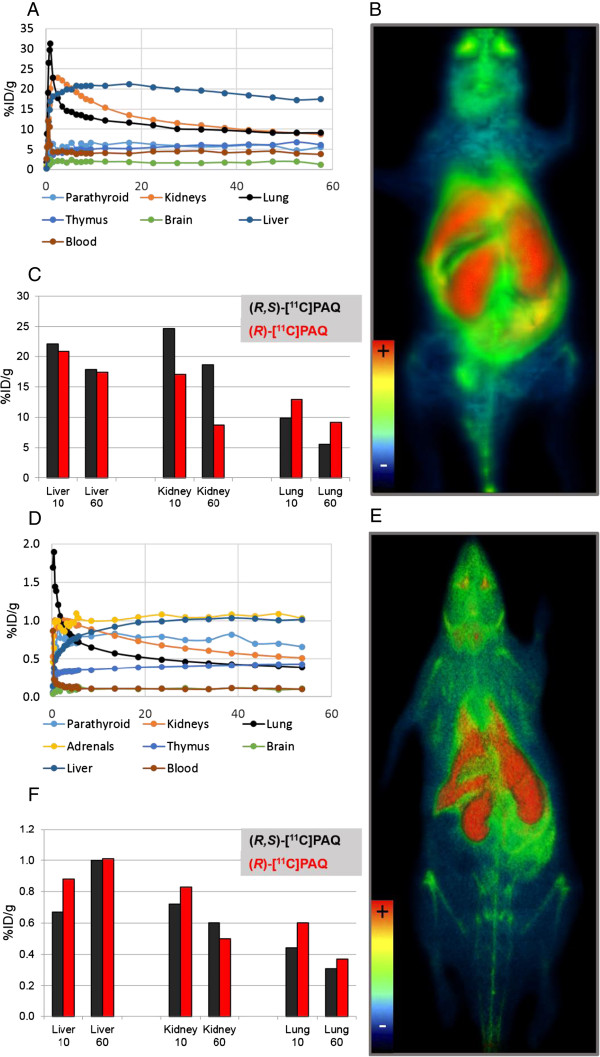
**Time activity curves and PET images.** Time activity curves and PET images (3-D volume rendering technique) over 60 min for a **(A**, **B)** WT mouse and **(D**, **E)** WT rat after injection of (*R*)-[^11^C]PAQ and a comparison of biodistributions for (*R*)-[^11^C]PAQ and (*R*,*S*)-[^11^C]PAQ in **(C)** mice and **(F)** rats. Organs and tissues examined are the same in both species except the adrenals, which could not be satisfactorily delineated in the mouse. (C) and (F) display the uptake in the liver, kidney and lung at early (9 to 11 min) and late (55 to 60 min) time points after injection of (*R*)-[^11^C]PAQ or (*R*,*S*)-[^11^C]PAQ in rat and mouse, respectively.

Regions of interest (ROIs) delineating organs and tissues were drawn on the images of the healthy animals used in the biodistribution studies (Figure 2). To estimate the blood levels of radioactivity, a ROI was drawn over the inferior vena cava 0 to 30 s post-injection. Assuming a tissue density of 1 g/ml, the radioactivity concentrations were divided by the administered activity to obtain ROI-derived percent injected dose per gram of tissue (%ID/g). Radioactivity concentrations (Bq/mL) were calculated automatically by calibrating against a phantom with a known concentration.

In the MMTV-PyMT mice, uptake of (*R*)-[^11^C]PAQ in the mammary gland tumour tissue was estimated by drawing a ROI over the upper right mammary gland. This position was chosen since the uptake here is least affected by spillover effects from other organs and tissues in these small animals. The mammary glands in the control animals were not visible in the PET images, and the ROIs were here placed in the area where the glands are normally located, an area with homogenously low normal uptake of [^11^C]PAQ. Lung uptake was assessed using a ROI created in the lungs at time frames that allowed good delineation from the heart and liver. Mean and maximum standardized uptake values (SUV_mean_ and SUV_max_) during the 20- to 60-min time period, when uptake had plateaued, were automatically calculated from the ROI.

Four animals (two MMTV-PyMT and two WT control animals) were injected with [^18^ F]FDG (0.3 to 0.4 MBq/g) 100 min after administration of (*R*)-[^11^C]PAQ and scanned for 40 min starting at time of injection. These animals were subsequently euthanized in the PET camera by an increased isoflurane concentration and moved to a 9.4 T MR scanner (Varian, Yarnton, UK) with efforts taken to minimize changes in body position. The animal holder with the mouse body was mounted in the scanner equipped with a volume coil of 40 mm inner diameter. The bodies were scanned using the spoiled 3-D gradient echo sequence (tr/te = 30/2.4 ms, flip angle = 11°). The matrix size was 512 × 192 × 192 covering a field of view of 80 × 30 × 30 mm^3^. The data were zero-filled to 512 × 256 × 256 before Fourier transformation to yield a voxel size of 156 × 117 × 117 μm^3^.

MRI data were acquired and reconstructed using the VnmrJ3.2 (Agilent Technologies, Santa Clara, CA, USA) software. Images were subsequently converted to Siemens/Concorde format via ImageJ (NIH, Bethesda, MD, USA) and AMIDE, imported to IRW and fused with the PET images for further analysis.

### Phosphor imaging

Lung and tumour tissue were excised from five MMTV-PyMT mice and cut into sections (25 μm) for PI and IF. Three mice were euthanized directly after the 60-min (*R*)-[^11^C]PAQ PET scan and two 30 min after tracer injection without undergoing PET. Lung tissue was fixated with OCT/PBS (1:1) (*n* = 4) through a needle in the trachea, and at least one of the mammary glands was removed. Tissues were snap frozen in isopentane, chilled with dry ice. Sections were cut from mammary glands with visible tumours and from the lung tissue of the MMTV-PyMT animals using a cryomicrotome (CM 3050S, Leica Microsystems, Wetzlar, Germany) and were placed on Superfrost Plus microscope slides (Menzel-Glaser, Braunschweig, Germany). The tissue sections were exposed for 60 min and subsequently read using a Typhoon 7000 FLA (GE Healthcare, Little Chalfront, UK) with the ImageQuantTL software (GE Healthcare). The digital images were analysed and processed using the ImageJ software (NIH, USA).

### Immunofluorescence

The same sections used for PI and additional sections from mammary gland and lung tissue from several animals were subjected to IF analysis. Sequential multiplex staining procedures were performed according to standard protocols using a combination of fluorescence-conjugated secondary antibodies and the tyramide signal amplification (TSA) method [15]. Briefly, sections were immersed in fixative (4% paraformaldehyde in 0.1 M phosphate buffer, pH 7.4). After washing in PBS, endogenous peroxidase activity was blocked by incubation in PBS containing 0.003% H_2_O_2_. Sections were incubated overnight with a rat anti-polyoma virus antibody (PyMT, 4°C, 1:5,000, clone NS1, ABCAM ab15085, Abcam, Cambridge, UK). Immunoreactivity was visualized using HRP-conjugated donkey anti-rat IgG (1:200, Jackson Immunoresearch, West Grove, PA, USA) and fluorescein-conjugated TSA (1:100, PerkinElmer). Sequential sections were incubated overnight in rat anti-CD31 (4°C, 1:100, clone; MEC13.3, BD Pharmingen, San Diego, CA, USA) followed by Alexa 594-conjugated donkey anti-rat IgG (1:200, Jackson Immunoresearch). Finally, sections were incubated overnight in rabbit anti-VEGFR2 (4°C, 1:2,000, Cell Signaling 55B11, Cell Signaling Technology, Danvers, MA, USA) and developed using HRP-conjugated swine anti-rabbit IgG (Dako, Glostrup, Denmark) and Cy5-conjugated TSA (1:100, PerkinElmer). Nuclei were stained using Hoechst 33342 (1:10,000).

Whole slides were captured using a 10× (Plan-APOCROMAT 10×/0.45) primary objective on a Vslide slide-scanning microscope (Metasystems, Altlußheim, Germany) equipped with filter sets for DAPI (EX350/50 - EM470/40), FITC (EX493/16 - EM527/30), Cy3.5 (EX581/10 - EM617/40) and Cy5 (EX630/20 - 647/long pass). Individual field-of-view images were stitched to produce images of entire lung sections with microscopic resolution. Images were analysed using the MetaViewer software (Metasystems).

## Results

### Biodistribution and metabolism of (*R*)-[^11^C]PAQ in wild-type rodents

High radioactivity uptakes were observed in the liver, kidney and lung with highest retention in the liver (Figure 2A,D). In the rat, noticeable uptakes were also found in the thymus and parathyroid glands, and highest overall uptake was in the adrenal glands. The whole-body PET of a WT mouse and a WT rat (Figure 2B,E) revealed that excretion of radioactivity to the bladder and intestines was limited during the 1-h scan. Distribution patterns of (*R*)-[^11^C]PAQ compared to the racemate are very similar, except in the mouse kidney where an appreciable difference in uptake was observed (Figure 2C,F).

The biodistribution and metabolism of racemate [^11^C]PAQ were previously reported in [10]. Here the *R*-stereoisomer behaviour was examined in a few healthy animals and compared with that of the racemate. In a confirmatory metabolism assay, rat arterial blood was sampled. At 30 and 60 min after injection, approximately 77% and 57%, respectively, of the radioactivity in the blood was intact (*R*)-[^11^C]PAQ. The small amount of radioactivity found in the urine was polar metabolites according to radio-HPLC. More detailed information can be found in Additional file 1.

### (*R*)-[^11^C]PAQ uptake in mammary glands and lung in MMTV-PyMT mice

**Figure 3:**
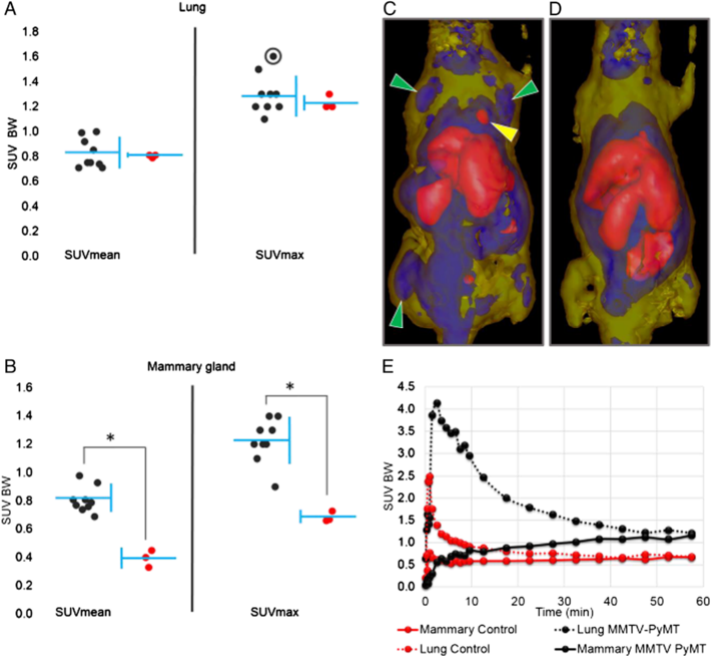
**Uptake in mammary glands and lung in MMTV-PyMT mice. (A)** Lung and **(B)** mammary gland uptake after injection of (*R*)-[^11^C]PAQ described as SUV_mean_ and SUV_max_ values. SUV values for MMTV-PyMT mice in black (*n* = 9) and WT in red (*n* = 3). All SUV values are calculated in relation to body weight (BW) from data summed from 20 to 60 min after i.v. administration of (*R*)-[^11^C]PAQ. Each individual is represented by a single dot; mean value and standard deviation of each group are indicated by the horizontal and vertical blue lines, respectively. Asterisk indicates a significant difference (unpaired *t* test, *p* < 0.005). **(C)** PET image of the MMTV-PyMT mouse (12 weeks) with highest SUV_max_ value in the lung; indicated with a circle in (A). **(D)** WT control mouse (13 weeks). The images are transparent isosurfaces with SUV > 1.5 = red colour, 0.7 < SUV < 1.4 = blue and 0.1 < SUV < 0.6 = yellow. Image data are collected for 60 min, starting at injection of (*R*)-[^11^C]PAQ. Green arrows indicate cancerous mammary glands, and the yellow arrow indicates an abnormal lung uptake. **(E)** TACs for lung and mammary gland tissue of the mice in **(C)** and **(D)**, respectively. TACs are derived from volume-standardized ROIs drawn over the area with highest uptake in each respective tissue of the mouse in **(C)** and the corresponding tissue of the mouse in **(D)**.

In all transgenic MMTV-PyMT mice (*n* = 9), the cancerous mammary glands could be clearly visualized with (*R*)-[^11^C]PAQ PET (Figure 3C). The mammary gland uptake in MMTV-PyMT mice was significantly (unpaired *t* test, *p* < 0.005) higher compared to that in the WT controls (Figure 3B), both when comparing mean and max SUV values. The lung uptake did not in general differ significantly between the groups (Figure 3A) although the two regions with the highest focal uptake were observed in the lungs of MMTV-PyMT mice (Figure 3C).

**Figure 4:**
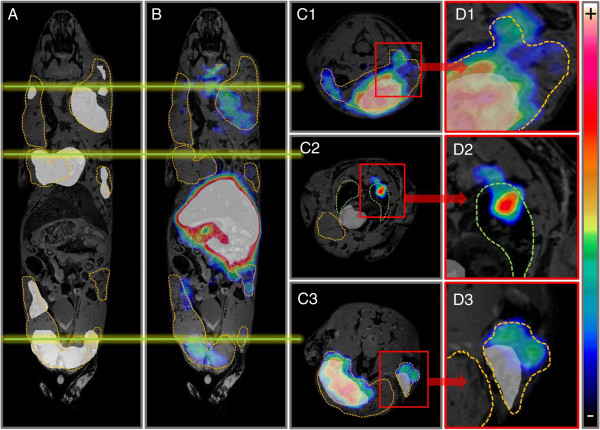
**MMTV-PyMT mouse scanned after injection with (*R*)-[**^**11**^**C]PAQ followed by [**^**18**^ **F]FDG 100 min later and MRI after euthanasia.** (*R*)-[^11^C]PAQ and [^18^ F]FDG images are summed over 60 and 40 min, respectively. Tumour areas are outlined with orange, and in **(C2)** and **(D2)** the lungs with green lines. **(A)** Areas with the highest [^18^ F]FDG uptake are shown as a white overlay over the MRI image. **(B)** (*R*)-[^11^C]PAQ images fused with the MRI image. The green lines drawn over (A) and (B) indicate the position of each transaxial image. **(C1-C3)** Transaxial images of the mouse with fused [^18^ F]FDG, (*R*)-[^11^C]PAQ and MRI images. **(D1-D3)** Areas of interest magnified, illustrating discrepancies in the uptake of the two tracers.

[^18^ F]FDG uptake partially overlapped the uptake of (*R*)-[^11^C]PAQ PET in mammary gland tissue in the MMTV-PyMT animals but was more homogenously distributed in the tumours (Figure 4). Uptake of [^18^ F]FDG could not be detected in the lung tissue in any of the animals. Some areas displaying obvious discrepancies in the uptake of [^18^ F]FDG and (*R*)-[^11^C]PAQ were also observed (Figure 4).

### *Ex vivo* immunofluorescence and phosphor imaging analyses

In order to corroborate that high levels of (*R*)-[^11^C]PAQ uptake were related to elevated expressions of VEGFR2, IF and PI analyses were performed on sections from cancerous mammary gland tissue of the MMTV-PyMT. Lung tissue, in which an increased uptake of (*R*)-[^11^C]PAQ was observed with PET, was analysed for the presence of metastatic activity.

**Figure 5:**
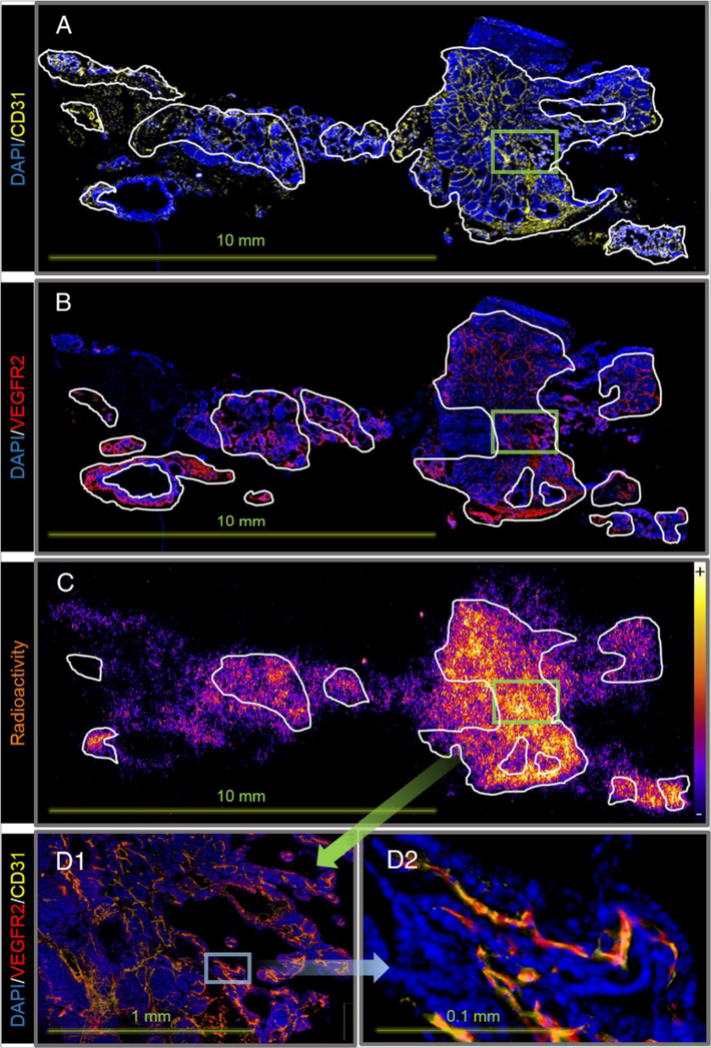
**Immunofluorescence and phosphor imaging of a representative mammary gland section from a 15-week-old MMTV-PyMT mouse.** Sectioning was performed 30 min after injection with (*R*)-[^11^C]PAQ. **(A)** Staining with DAPI (blue) and CD31 antibodies (yellow); the most intensely CD31-stained areas are outlined. **(B)** Staining with DAPI and VEGFR2 antibodies (red); the most intensely VEGFR2-stained areas are outlined. **(C)** Phosphor imaging displaying radioactivity distributions with an overlay (outlined) of areas with overlapping high VEGFR2 and CD31 expressions. **(D1)** Magnification of a region with high uptake of radioactivity (indicated with a green square in **(A)** to **(C)**) showing the corresponding image with IF where the staining of CD31 and VEGFR2 are merged. **(D2)** Further magnification indicated by the blue square in **(D1)** showing VEGFR2 and CD31 co-localization in VEGFR2-positive blood vessels.

The radioactivity distribution (Figure 5C) in the section correlated well with that of the VEGFR2 expression (Figure 5B). When the most intensively stained areas of VEGFR2 and CD31 overlaid the phosphor image, correlation is even more precise (the radioactive hotspots in Figure 5D1,D2). Radioactivity uptake was observed in areas with high VEGFR2 but low CD31 expressions, but is lower than in areas where both are co-localized. Relatively low uptake of radioactivity was observed in areas with high CD31 and low VEGFR2 expressions.

**Figure 6:**
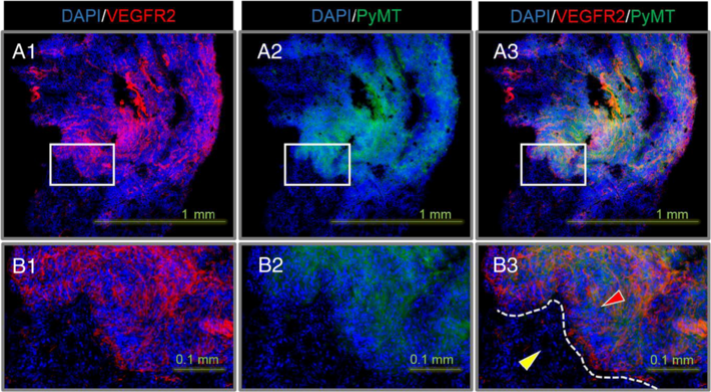
**IF staining of a lung section from the MMTV-PyMT mouse in Figure **4**.** DAPI (blue), VEGFR2 (red) and PyMT (green). **(A1-A3)** Sections containing a metastasis with a diameter of approximately 1 mm. **(B1-B3)** Magnification of the corresponding area indicated with a square in (A). **(B3)** Red and yellow arrows indicate metastasis and normal lung tissue, respectively. The border between the tissues is outlined in white.

The metastases in the stained lung sections were characterized by a higher tissue density compared to normal lung tissue (Figure 6), intense VEGFR2 staining (Figure 6A1,B1) and a marked PyMT-staining (Figure 6A2,B2). The PyMT-specific antibody and the higher VEGFR2 staining correlate well and show that neoangiogenesis is present everywhere within the metastatic growth (Figure 6A3). A distinct border can be seen between the metastatic tissue and the normal lung tissue (Figure 6B3). This metastasis could be observed in approximately 30 sequential sections (25 μm). The section in Figure 6 is representative for the observations.

## Discussion

This study introduces the *R*-isomer of [^11^C]PAQ as a noninvasive PET imaging biomarker for VEGFR2 expression. (*R*)-[^11^C]PAQ demonstrates favourable imaging properties, including limited metabolism, rapid clearance from the blood and rapid accumulation in the tumour tissue. Using the MMTV-PyMT model of metastatic breast cancer in mice, we have shown that (*R*)-[^11^C]PAQ can visualize lesions with areas of ongoing neovascularization. *Ex vivo* analysis corroborated that tracer uptake in cancerous mammary gland tissue is highest in areas with high expressions of VEGFR2 co-expressed with CD31. Lung regions displaying metastatic growth were also detected using (*R*)-[^11^C]PAQ PET.

The *R*-isomer of [^11^C]PAQ was developed because of its high selectivity for VEGFR2 and also to minimize potential pharmacokinetic complications when using the two enantiomers simultaneously. The importance of stereoisomerism and possible problems associated with racemic PET tracers has been discussed in [16], and it was concluded that only pure stereoisomers should be used for quantitative PET imaging. Although the review focused on PET tracers for brain studies, several of the pharmacokinetic parameters also apply for PET studies in peripheral tissue. In our comparisons of the biodistributions of (*R*)-[^11^C]PAQ and (*R,S*)-[^11^C]PAQ, only slight differences were observed between species regarding lung and liver uptake, while the uptake in the kidney showed a larger difference in mouse (Figure 2C,F). The *R-* and *S*-isomers may differ in nonspecific binding, metabolic stability and distribution volumes, and these differences can also be species dependent. Translation to applications in human is therefore most likely improved when only the more selective *R*-isomer is used.

VEGFR2 is abundantly expressed in the liver, lung and kidney in the adult mouse. In secretory organs such as the adrenal gland, the concentrations of VEGF are relatively high and it is implied that the expressions of VEGFR2 follow that of its ligand and are elevated as well [17]. The presence of VEGFR2 might therefore contribute to the high uptake of (*R*)-[^11^C]PAQ observed in these organs (Figure 2A,D).

As shown in Figure 5, the uptake of radioactivity and the VEGFR2 expressions overlap in many areas, with the best correlation in areas having both high VEGFR2 expressions and a higher microvascular density, indicated by CD31 staining. This indicates that the tracer binds to VEGFR2 in tumour tissue and that the degree of tracer binding will also be related to the vascularization of the tissue. On a microlevel, the importance of delivery of the tracer to areas containing VEGFR2 is illustrated and shows that (*R*)-[^11^C]PAQ is capable of visualizing tissue with sufficient perfusion and ongoing angiogenesis. On a macro PET imaging level, lesions and metastasis with increased angiogenesis and neovascularization could be readily visualized in a noninvasive fashion, as shown in Figure 3.

Serial PET examinations with (*R*)-[^11^C]PAQ followed by [^18^ F]FDG were performed to investigate whether this new imaging biomarker could provide added information over the clinically widely used [^18^ F]FDG. Both tracers accumulated in the cancerous mammary gland tissue, though in some instances with an obvious difference in uptake patterns (Figure 4C3,D3). These differences may be due to angiogenesis occurring in specific areas of the tumour [18], while cells with high glucose utilization, indicated by high [^18^ F]FDG uptake, are a general feature and they are more evenly distributed within the tumour. In the MMTV-PyMT model, multiple tumours at different stages of tumour progression often develop simultaneously within the mammary glands, which could further explain the more heterogeneous uptake pattern of (*R*)-[^11^C]PAQ observed in both Figures 4 and 5. The MMTV-PyMT mouse examined with PET and MRI (Figure 4) had a marked uptake of (*R*)-[^11^C]PAQ but not [^18^ F]-FDG in the lung. The IF results for lung sections from this animal shown in Figure 6 confirm metastatic and highly angiogenic activity in this area. These novel findings imply that (*R*)-[^11^C]PAQ may be better than the most widely used imaging biomarker in oncology, [^18^ F]FDG, in visualizing early metastatic growth in, for example, lung tissue.

Several small-molecule PET tracers targeting the tyrosine kinase domain of the VEGFR2 have been synthesized and are reviewed in [19,20]. Recently, ZD6474 has been labelled with carbon-11, but *in vivo* results have not yet been published [21]. In addition, a fluorine-18-labelled PAQ analogue has been evaluated, with *ex vivo* binding to resected tissue from human glioblastoma tumours shown [22]. Other approaches to visualize angiogenesis with PET are protein-based VEGFR ligands [23,24] and arginylglycylaspartic acid (RGD) peptides targeting integrin αVβ3 [25]. Promising imaging results have also been reported for [^89^Zr]bevacizumab which binds to VEGF itself. An increased uptake compared to labelled nonspecific control proteins was observed in angiogenic tissue, and the uptake levels were shown to correlate to angiogenic activity during treatment and to the efficacy of anti-tumour drugs [26,27]. PET tracers toward these and other targets may potentially provide complementary information about angiogenic processes and can be useful for stratifying patients for therapies directed at these specific targets.

To be clinically useful, a PET tracer must not only have good prognostic value but also be relatively easy to obtain. The carbon-11 labelling procedure used in the synthesis of (*R*)-[^11^C]PAQ is based on radiochemistry that is readily performed in most PET facilities. The pharmacokinetics examined in rodents indicates fast localization of the tracer to its intended target (favourable for rapid ^11^C-based imaging) and limited metabolism with high levels of intact tracer during the initial tissue accumulation phase. In addition, (*R*)-[^11^C]PAQ has a favourable biodistribution for cancer studies in most parts of the body, except possibly the liver and kidneys due to high normal uptakes in these organs. Rapid distribution from the blood and a tendency to plateau in tumours relatively quickly could favour protocols for injecting and later moving to the PET scanner for a shorter turnaround time. Since the carbon-11 isotope has a short half-life of approximately 20 min, same-day imaging of the patient with another PET tracer could be performed to examine different biochemical and biological characteristics of the lesions. All these factors indicate that (*R*)-[^11^C]PAQ may be a promising PET imaging biomarker also in the clinical setting.

## Conclusion

(*R*)-[^11^C]PAQ is a promising tracer that can be used to visualize elevated levels of angiogenesis in primary solid tumours and likely also in tissues with metastatic growth. The rapid accumulation of tracer in tumour tissue provides good visualization within 60 min after administration and is consistent with the short-lived carbon-11 radionuclide. The biodistribution of the tracer is favourable for detection and characterization of malignant tissue particularly in the head and neck and thorax regions in rodents. We suggest that the tracer should be further evaluated preclinically and subsequently in humans, with focus on establishing protocols to find and assess local areas with angiogenic activity. Based on our preclinical findings, we believe that a clinical use of (*R*)-[^11^C]PAQ PET could be a very important diagnostic complement for characterization of disease and in selecting patients for anti-angiogenic therapy.

## Competing interests

The authors declare that they have no competing interests.

## Authors’ contributions

ES, SSE, LH, TT and HR conceived and coordinated the study and drafted the manuscript. ES and JT performed the synthesis of the precursor, reference compound and (*R*)-[^11^C]PAQ. LL and HR performed the PET examinations and *ex vivo* studies. LL and ES conducted the *in vivo* metabolism study. PD carried out the MRI examinations. JM performed the immunohistochemistry studies. All authors read and approved the final manuscript.

## Supplementary Material

Additional file 1 **Synthesis of the *N*-desmethyl precursor and (*R*)-[**^**11**^**C]PAQ.** Click here for file
