# Supplementary material for: Visualization of angiogenesis during cancer development in the polyoma middle T breast cancer model: molecular imaging with (R)-[11C]PAQ
Source: EJNMMI Res. 2014 Mar 26;4:17. doi: 10.1186/2191-219X-4-17 (PMC3986910; doi:10.1186/2191-219X-4-17)
Supplement: Additional file 1 — Synthesis of the N-desmethyl precursor and (R)-[11C]PAQ. [file 2191-219X-4-17-S1.docx]

**SUPPLEMENTARY MATERIALS**

**Synthesis of the *N*-desmethyl precursor and (*R*)-[^11^C]PAQ**


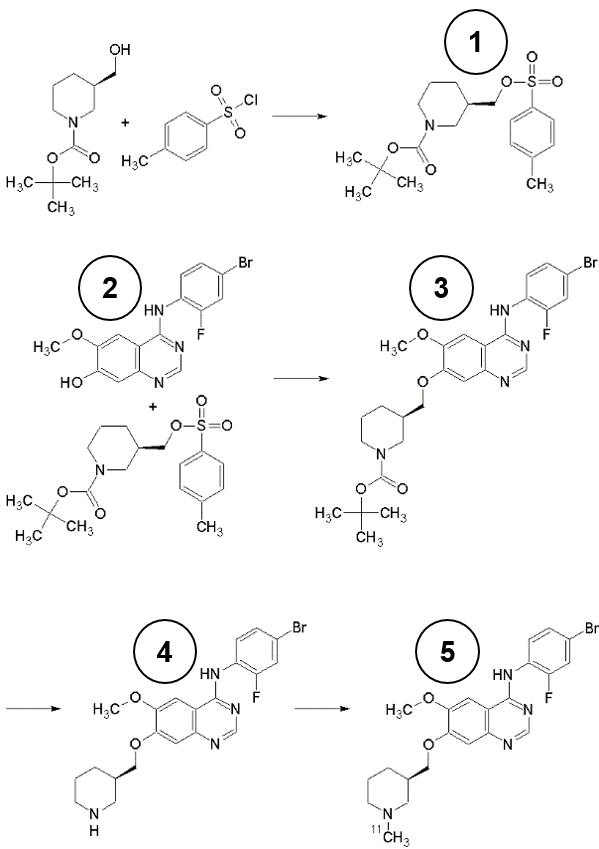


Figure 1. Route of synthesis for N-desmethyl precursor and (R)-[^11^C]PAQ.
**1:** (*R*)-tert-butyl 3-({[(4-methylphenyl)sulfonyl]oxy}methyl)piperidine-1-carboxylate
**2:** 4-(4-bromo-2-fluorophenylamino)-6-methoxyquinazolin-7-ol
**3:** (*R*)-tert-butyl 3-((4-(4-bromo-2-fluorophenylamino)-6-methoxyquinazolin-7-yloxy) methyl)piperidine-1-carboxylate
**4:** (*R*)-N-(4-bromo-2-fluorophenyl)-6-methoxy-7-{(3-piperidinyl)methoxy}-4-quinazolinamine: (desmethyl *R*-PAQ)
**5:**(*R*)-N-(4-bromo-2-fluorophenyl)-6-methoxy-7-{(1-[^11^C]methyl-3-piperidinyl)methoxy}-4-quinazolinamine: ((*R*)-[^11^C]PAQ). Molecular weight = 475.35 g/mol.

**Preparation of 1:** To a solution of pyridine (1 ml), triethylamine (2 ml) and (R) *tert*- butyl-3-(hydroxymethyl)piperidine-1-carboxylate (480 mg) at 0°C, was added tosyl chloride (600mg). The red solution was stirred at 0°C ice for 1h then at 23°C for 17 h at which time the reaction was complete according to TLC. The solution was added to ice and extracted with dichloromethane (20 ml). Solvents were removed under reduced pressure and the product purified on flash-chromatography (toluene: ethyl acetate 19:1) to give 500 mg of 1 (61% yield).

**2** was purchased from Sun Biochem Inc. CO, USA.

**Preparation of 3:** 1 (200 mg) and 2 (175 mg) were dissolved in dimethylformamide (3 ml) at room temperature. Finely ground K_2_CO_3_ (210 mg) was added. Temperature was raised to 105 °C and the reaction was complete according to TLC after 4 h. Brine was added and the mixture was cooled at -20°C for 1 h. The resulting yellow-white solids were removed by filtration and dissolved in ethyl acetate, washed with water and dried over MgSO_4_. The ethyl acetate was evaporated under reduced pressure to afford 280 mg of yellow sticky material which was further purified with flash-chromatography using a gradient with toluene:ethyl acetate 1:2 🡪 7:3 on a reversed phase silica column to afford 170 mg of 3 (63% yield) as an amorphous liquid which crystallized over 24 h in room temperature.

**Preparation of 4:** 170 mg of 3 was dissolved in dichloromethane (3 ml) and trichloroacetic acid (0.4 ml) was added and allowed to react for 3 h at room temperature at which time the reaction was complete according to TLC. After evaporation, 140 mg (99% yield) of the desired product was obtained as an off white solid. This product was used in the radiosynthesis without further purification. The chemical and optical purity of the product (>99%) was confirmed by HPLC (Daicel Chiralpak 127; isopropyl alcohol:hexane 1:9 v/v). The (R,S)-precursor was used as reference on the HPLC-system.

**Preparation of 5:** ((R)-[^11^C]PAQ) was synthesized and purified using an automated synthesis module (Tracerlab FX C Pro, General Electric Medical Systems AB). The N-desmethyl precursor (1 mg) and K_2_CO_3_ (10 mg) in dimethylfomramide (0.4 ml) were mixed and subsequently filtered through a syringe filter (PVDF, 4 mm, National Scientific company) into a reaction vial placed in the synthesis module. Cyclotron (PETtrace, General Electric Medical Systems AB)-produced [^11^C]methane was delivered to the module and converted to [^11^C]methyl iodide. The desired radiotracer was isolated from preparative HPLC on a µ-Bondapak C18 column (Waters) (300 x 7.8 mm, 10 µm; CH_3_CN:0.05 M NH_4_OAc 70:30 v/v) followed by solid phase extraction on a C18 SepPak Plus (Waters), acidification with 0.02 M H_3_PO_4_ and elution with ethanol (99%). Prior to administration the product solution was diluted with saline to achieve a maximum ethanol content of 10% (v/v).

All solvents and reagents used in the synthesis were commercially available. The synthesis procedures were repeated with similar yields.

**Metabolite assay**

*
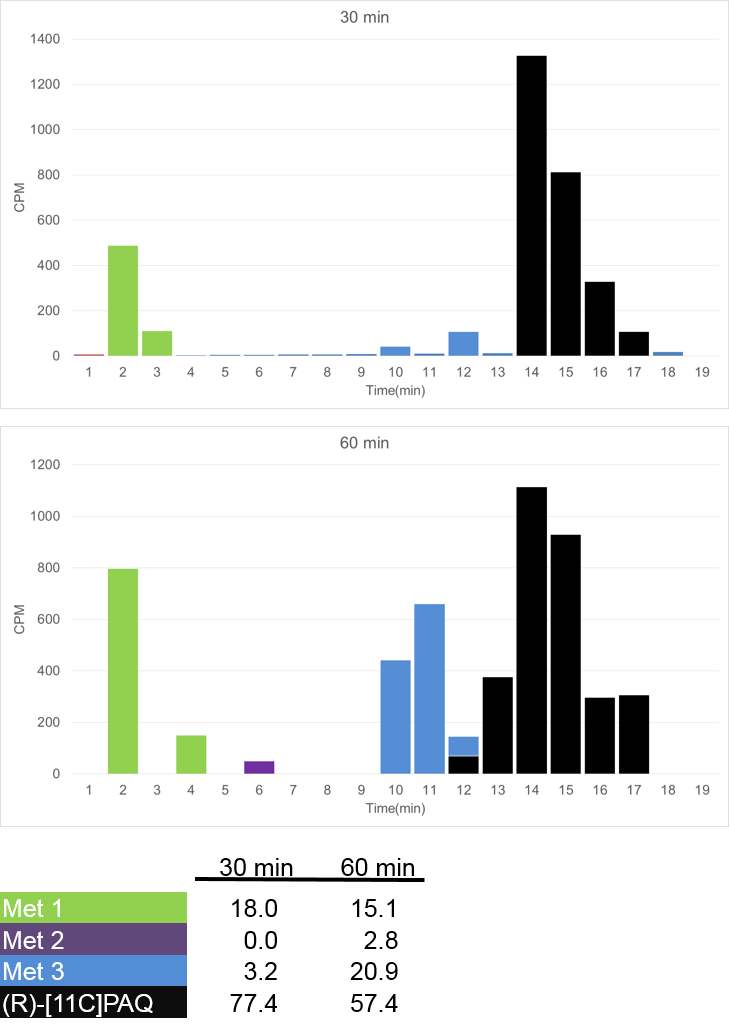
*

Figure 2. Metabolites detected in plasma from blood sampled 30 and 60 min after injection with (R)-[^11^C]PAQ in a male rat. The table describes the percentage of total radioactivity of the different metabolites and (R)-[^11^C]PAQ) in each sample. (*R*)-PAQ) was used as non-labelled reference and it co-eluted with the radioactivity peaks with black color.
